# Complete Genome Sequence of a Lineage I Peste des Petits Ruminants Virus Isolated in 1969 in West Africa

**DOI:** 10.1128/genomeA.00381-15

**Published:** 2015-05-07

**Authors:** William G. Dundon, Daojin Yu, Modou Moustapha Lô, Angelika Loitsch, Mariame Diop, Adama Diallo

**Affiliations:** a Joint FAO/IAEA Division of Nuclear Techniques in Food and Agriculture, Department of Nuclear Sciences and Applications, Animal Production and Health Laboratory, International Atomic Energy Agency, Vienna, Austria; b College of Animal Science, Fujian Agriculture and Forestry University, Fuzhou, People’s Republic of China; c Laboratoire de Virologie ISRA/LNERV, Dakar, Senegal; d Institute for Veterinary Disease Control, Austrian Agency for Health and Food Safety, Mödling, Austria

## Abstract

We report the complete genome sequence of a lineage I peste des petits ruminants virus (E32/1969) isolated in a Senegalese laboratory in 1969. This is the earliest peste des petits ruminants virus of any lineage sequenced to date and only the second lineage I virus available in public databases.

## GENOME ANNOUNCEMENT

Sheep and goats contribute considerably to the cash income and nutrition of small farmers in many countries in Africa and Asia, so the control of the highly infectious transboundary viral disease of small ruminants, peste des petits ruminants (PPR), is considered an essential element in the fight for global food security and poverty alleviation. PPR is caused by the peste des petits ruminants virus (PPRV), which is a member of the genus *Morbillivirus* within the family *Paramyxoviridae* (1). It is a nonsegmented, negative, single-stranded RNA virus that encodes six structural proteins, namely, the nucleocapsid protein (N), phosphoprotein (P), matrix protein (M), fusion protein, (F), hemagglutinin protein (H), RNA-dependent RNA polymerase (L), and two nonstructural proteins (V and C). PPRVs have been classified into four genetic lineages (I–IV). Lineage IV is the only lineage circulating in Asian countries, while all four lineages have been found in Africa (2). To date, there are 23 full-genome sequences available in public databases representing the four lineages, but only one of those is from lineage I.

In March 2013 a lyophilized specimen was shipped by the Laboratoire de Virologie ISRA/LNERV, Dakar, Senegal, to the Animal Production and Health Laboratory, Vienna, for further characterization. The specimen dates back to 1969, and although the original sample from which the specimen was derived is believed to have been collected in Senegal, the sample’s exact origin is unclear. What is known, however, is that five goats were experimentally infected in June 1969 using the original sample. All of the animals died following classical PPR symptoms—namely, depression, diarrhea, respiratory difficulties, and serous and mucopurulent nasal discharges. The lungs of the dead animals were ground and passaged three times in lamb kidney cells. Aliquots (1 mL) of the infected cultures were then lyophilized in September 1969. On arrival in Vienna, the lyophilized specimen was resuspended in 1 mL of Dulbecco’s modified Eagle’s medium (high glucose medium) plus antibiotics, and 100 µL was used to infect CHS 20 cells (3). A cytopathic effect was observed after 5 days. RNA was extracted directly from the infected cells and analyzed by RT-PCR for the presence of PPRV RNA (4). Phylogenetic analysis of the sequence of the amplicon generated revealed that the virus belonged to lineage I. The RNA was then subjected to full-genome sequencing as described previously (5, 6). The organization of the PPRV E32/1969 genome (15,948 bp) was identical to that seen for other PPRVs with a 107-nucleotide genome promoter region at the 3′ end followed by the transcription units for the N, P, M, F, H, and L proteins and the antigenome promoter at the 5′ end. The genome has the highest nucleotide sequence identity (97.1%) with the lineage I virus ICV89 (EU267273) (7) and the lowest identity (89.3%) with the lineage III virus KN5/2011 (KM463083) (6).

This is the earliest PPRV genome sequenced to date and only the second lineage I virus available in GenBank. The sequence provides important information to those working on the molecular evolution of this important transboundary disease.

### Nucleotide sequence accession number.

The complete genome sequence of E32/1969 has been deposited in GenBank under the accession number KP789375.
